# Disclosure of Investigators' Recruitment Performance in Multicenter Clinical Trials: A Further Step for Research Transparency

**DOI:** 10.1371/journal.pmed.1001149

**Published:** 2011-12-27

**Authors:** Rafael Dal-Ré, David Moher, Christian Gluud, Shaun Treweek, Jacques Demotes-Mainard, Xavier Carné

**Affiliations:** 1 Early Detection Research Program, Alzheimer's Disease, Pasqual Maragall Foundation, Barcelona, Spain; 2 Clinical Epidemiology Program, Ottawa Hospital Research Institute; Department of Epidemiology and Community Medicine, Faculty of Medicine, University of Ottawa, Ottawa, Canada; 3 Copenhagen Trial Unit, Centre for Clinical Intervention Research, Rigshospitalet, Copenhagen University Hospital, Copenhagen, Denmark; 4 Population Health Sciences, School of Medicine, University of Dundee and Tayside Clinical Trials Unit, Dundee, United Kingdom; 5 INSERM, Paris, France; 6 European Clinical Research Infrastructures Network (ECRIN), Paris, France; 7 Clinical Pharmacology Service, Hospital Clinic, University of Barcelona, Barcelona, Spain

## Abstract

Rafael Dal-Ré and colleagues argue that the recruitment targets and performance of all site investigators in multi-centre clinical trials should be disclosed in trial registration sites before a trial starts, and when it ends.

**Summary Points**

- Many clinical trials are terminated before reaching the sample size needed to test the trials' hypotheses owing to poor recruitment.
- Registries, such as ClinicalTrials.gov, provide information on the main features of a multicenter clinical trial (MCT) to the general public.
- Site investigators are key to the success of MCTs; however, information on their recruitment performance is not publicly available.
- We propose that sponsors should disclose the recruitment targets of all site investigators on ClinicalTrials.gov before a trial starts as well as their final recruitment. Information on issues that could have affected recruitment should also be provided.
- This information will be of interest to different stakeholders such as patient organizations, sponsors, and MCT networks.
- Disclosing all site investigators' recruitment figures could prompt queries to the sponsor from the scientific community about regional subgroup analyses, to assess if ethnic or standard-of-care differences have an impact on treatment outcomes.

## Transparency: A Fundamental Social Obligation for Clinical Research

After 60 years devoted to enhancing the methodology and ethics in clinical research, the last decade has been crucial to the scientific community in refining the transparency on conducting clinical trials (CTs), from their inception to the publication of results. A myriad of articles have been published on the design, conduct, conflicts of interest, reporting, and publication of CTs. Now a responsible investigator (or sponsor) involved in the design and conduct of a CT must, in addition to obtaining research ethics committee (REC) and, in many countries, regulatory approvals, register the protocol in a publicly accessible registry before recruiting the first participant and publish (or otherwise make publicly available) the results obtained [1]–[4]. There are many CT registries, 14 of which are included on the WHO International Clinical Trials Registry Platform [5]. ClinicalTrials.gov [6], the largest with more than 90,000 CTs from around the world, provides a substantial amount of information to anybody interested in a trial. However, an important piece of information is lacking: recruitment information for each site participating in the CT. We believe in the benefits of increased transparency in all phases of clinical research, since it has been proven not only to build trust, but also to be one of the best tools to reduce bias, to improve credibility of the results, and to foster the efficient management of trials.

## Recruitment: A Crucial Aspect in Conducting a Clinical Trial

A critical aspect to carefully consider in the planning of a CT is the number of participants to be recruited in a specified time period and to outline contingency plans if problems in recruitment arise. It is well known that recruiting participants can be a significant burden and is frequently the most difficult task in conducting a CT. Failure to reach the planned sample size within the agreed time frame and funding is common. Poor recruitment is a waste of resources and a potential abuse of participant goodwill. In the UK, only 31% of CTs sponsored by the Medical Research Council and the Health Technology Assessment Programme achieved their original recruitment target; furthermore, 55% did not reach the revised target [7]. In 333 concluded UK public and charity-sponsored cancer trials that were started between 1971 and 2000, only 48% reached the planned sample size, whereas 20% of the CTs recruited less than 25% of the planned sample size [8]. Recent US data showed that among 180 National Cancer Institute (NCI) Cancer Evaluation Program-sponsored CTs, activated between 2000 to 2004 and closed to accrual, 36% and 62% of phase 2 and 3 trials, respectively, did not attain their recruitment goals [9].

This pattern in publicly funded trials is also occurring in industry-sponsored trials. For example, in an industry-sponsored multicenter clinical trial (MCT) on asthma conducted in 11 countries, only 35% of sites succeeded (or exceeded) in recruiting the committed number of patients [10]. In fact, some companies assume in the planning phase that up to 25% of the sites in a MCT will never enroll a single patient [11], which emphasizes the difficulty of recruiting participants. Reports from RECs also found a significant percentage of CTs with poor recruitment [12]–[14]. Overestimating the pool of eligible patients or the recruitment rate (known as “Lasagna's law”) occurs even though Good Clinical Practice guidelines state that investigators “should be able to demonstrate (e.g., based on retrospective data) a potential for recruiting the required number of suitable subjects within the agreed recruitment period,” and that “the investigator should have sufficient time to properly conduct and complete the trial within the agreed trial period” [15]. Recently a number of reviews on interventions to improve CT recruitment have been published [16],[17].

Except for some early proof-of-concept CTs, a sample size estimation should be conducted and reported in the trial protocol for all randomized CTs—something that is frequently lacking [18],[19]—or a rationale for not doing so should be included in the study protocol. Data show that in up to 66% [19] to 75% [20] of CT reports, there is no reporting of the sample size estimation.

In planning a CT, sample size estimation will trigger many decisions about the number of recruitment sites (and even countries) required to successfully accrue participants. If the number of participants is such that the research question cannot be properly answered, the trial may risk becoming scientifically useless and ethically unacceptable: the CT will bring burdens and risks (e.g., adverse effects) to participants, with no medical benefit for future patients because of its lack of scientific usefulness (e.g., no statistically significant difference detected between treatment groups). The question that follows is: what about the many CTs that never recruit the number of participants needed to appropriately test the underlying hypothesis? Is this not a (subtle) way of not fulfilling the investigator–participant contract inherent in a CT? If the results are not published in peer-reviewed journals, the investigator or the sponsor should make the results available through a private registry (e.g., company website) and provide a link to the website through a public registry (e.g., ClinicalTrials.gov). If the obligation to making the results publicly available is fulfilled, a CT that has not recruited the number of participants needed is underpowered and less informative from the perspective of the individual CT; nonetheless, the data, may be of scientific use if they are used to conduct meta-analyses.

## The Proposal: To Provide Information on All Site Principal Investigators' Recruitment Performance

Recruitment performance of clinical centers and investigators varies significantly [10], in both public- and private-sponsored CTs, although most drug MCTs are sponsored by industry [21]. Many investigators will participate in a number of trials in their professional careers, sometimes even simultaneously for the same condition. Through public registries, such as ClinicalTrials.gov, access to information is made available on the scientific rationale, methods, sponsorship, scientific leadership, calendar, contacts, and locations. Although currently available CT registration enhances transparency, deficiencies have been found that undermine the full potential usefulness of registries [22]. We believe that recruiting sites' principal investigators should be accountable for the conduct and outcome of their trial. This should be regarded similarly to the current practice of publishing MCT results in a manuscript with all recruiting researchers included as members of the “study group.” Hence, we propose not only to disclose the names, contact details, and affiliations of all sites' recruiting investigators in the registry before trial start, something that is currently being provided by a variety of sponsors [6], but also to reveal their recruitment commitment and the number of recruited participants (Table 1).

**Table 1:** Key elements of principal investigators at each recruiting site involved in multicenter clinical trials to be disclosed in a publicly open clinical trial registry such as ClinicalTrials.gov.

Element	Description
1. Name, contact details (e.g., email address, phone), and affiliation (department, institution, city, country)^a^	—
2. Site recruitment status: “not yet recruiting,” “active, but not recruiting,” “recruiting,” “completed,” or “withdrawn”	—
3. Recruitment commitment: number of participants to be recruited.	This figure refers to those participants who are to be randomized.
4. Actual recruitment: number of participants actually randomized.	—
5. Non-investigator–related issues impacting clinical trial recruitment:	Delays due to REC/institutional review board reasons
	Delays due to regulatory reasons
	Delays due to center–sponsor contract negotiations
	Issues with sample dispatch
	Halt randomization of participants included in the prerandomization period^b^
	Early termination of the clinical trial^c^
	Other (specify)
6. Unexpected investigator-related issues impacting clinical trial recruitment:	Health issue
	Other (specify)

If there is more than one principal investigator in a site all the information should be provided on all of them.

a Multiple locations may be specified on ClinicalTrials.gov. Currently, when provided, the name (and contact details) of a contact person (not necessarily the site principal investigator) is usually given. On the other hand, there are examples in which contact details of all site principal investigators are provided: these are multicenter clinical trials sponsored by, for instance, nonprofit organizations (e.g., NCT01264445), governmental institutions (e.g., NCT01108614), research foundations (e.g., NCT00662220), industry (e.g., NCT00944905), universities (e.g., NCT00973154), collaborative groups (e.g., NCT00209209), or hospitals (e.g., NCT00756600) [6].

b Due to early achievement of the number of (randomized) participants needed in the clinical trial.

c When the trial is terminated, for whatever reason, before completing the expected recruitment. Currently ClinicalTrials.gov allows inclusion of information regarding suspended, terminated, or withdrawn studies providing “a brief explanation of why the study has been halted or terminated. If desired, use brief summary or detailed description to provide additional information. (Limit: 160 characters)” [6].

It should be highlighted that some sponsors do currently provide information on the recruitment status of all sites involved in an MCT. Thus, in ClinicalTrials.gov [6], near the site principal investigator contact details, the recruitment status is sometimes provided as “not yet recruiting,” “active, but not recruiting,” “recruiting,” “completed,” or “withdrawn”. We propose for a given site, that if the trial has more than one recruiting principal investigator, then information for all recruiting investigators should be provided. This information will allow readers to assess the performance of each site principal investigator. This information may be of interest to the REC (or institutional review board) assessing the qualifications of a given researcher (and CT underway or committed in the same population) when reviewing a CT protocol [15], and to patient organizations, hospital and university managers, CT networks and collaborative groups, public and private sponsors, and health authorities. Thus, sponsors willing to start a CT may wish to recruit investigators with a track record of fulfilling their commitments, hospital or university managers may want data on the performance of their investigators in an activity that can impact their institutions' prestige, and patient organizations may want to know the investigators who actually recruit most participants in CTs in their area of interest. Furthermore, since it is well known that both ethnic and standard-of-care differences can impact treatment outcomes in different geographical regions [23]–[25], having access to the percentage of participants coming from different countries (and, hence, regions) could prompt queries to the sponsor from experts about regional subgroup analyses. In addition, providing recruitment data could be of interest to researchers conducting prospective meta-analysis and individual patient data meta-analysis to examine specific outcomes and harms. Participants in a trial have the right to be informed on the results obtained [3]: if an MCT is not concluded because of recruitment issues, recruiting site principal investigators could transparently inform trial participants wishing to know which centers did not deliver as expected, because the data are available on the registry. Finally, misconduct and fraud in clinical research does happen [26],[27]. Full transparency regarding recruitment goals and results should help to reduce the risk of misconduct and fraud.

## How to Comply with the Proposal: The Practicalities

Registering investigators' recruitment performance information in MCTs should be straightforward, since a record of all research sites is always regularly up-dated (on a real-time enrollment basis in many trials) by the sponsor or the coordinating center. Hence, this proposal represents a modest burden on sponsors. It is reasonable to believe that the fact of having these data publicly disclosed will encourage site recruiting investigators to ask the sponsor to update the information; at the end of the day, each investigator should take responsibility for his/her own reputation in relation to patients, institution, and other researchers. At the very least, the recruitment final data per site principal investigator should be provided when recruitment of participants has been concluded (having achieved or not achieved the trial's target number of participants). In any case, information regarding recruitment per site is usually included in the MCT final report and when reporting industry-sponsored trials results to regulatory agencies [28].

Importantly, once the initial list of recruiting centers and site principal investigators (and, following our proposal, their recruitment targets) is provided, the list will not usually change unless (a) an investigator is asked to stop his/her contribution to the trial because of a lack of recruitment; in this case, the sponsor should include the appropriate information on the registry, or (b) new recruiting centers or investigators are added to the original list, most likely as a contingency plan to enhance the trial's poor recruiting rate to date. After the initial target between the sponsor and each site investigator is agreed upon, further agreements should be registered; a record of these agreements will be available on the registry's archive site thanks to the audit trail [6].

Any related issue that could affect recruitment in a site should be disclosed, thus providing the reader with the necessary information to understand what happened during the recruitment period of the trial. Among the non-site investigator related issues, and to facilitate submission of information, we propose that this information should be classified in a number of “closed” reasons, as shown in Table 1. Similarly, any unexpected investigator-related reason that could impact his/her recruitment performance should be recorded. We suggest only a “closed” reason (“health issues”) in this section.

## Conclusion

Since all parties involved (from research-funding agencies and regulatory agencies to journal editors and industry) are asking for transparency in CTs [4],[29], we propose that its level should be taken beyond the current limits. We acknowledge that several critical issues need to be addressed in the near future to enhance CT transparency. Among these are the urgent need for sponsors to provide complete and accurate information as required by public registries [22] as well as access to trial protocols and raw databases [30]. In this respect, we propose that all MCTs registered in publicly available registries, such as ClinicalTrials.gov [6], should provide detailed information about the names, contact details, recruitment commitments, and recruitment deliveries of all site principal investigators involved. This information will make them accountable for the MCT performance not only to the sponsor, but also to all participating patients or healthy volunteers and recruiting investigators, all other interested researchers in the field, and, ultimately, society as a whole. In fact, disclosing the recruitment performance of all site recruiting principal investigators is a small but relevant part of an MCT's raw data; they must be considered as belonging to the scientific study information that should be openly shared, something that is currently being promoted by private and public funding organizations [31]. Similar to other initiatives established to improve transparency, such as the CONSORT statement—although this was enforced by journal editors—moves by sponsors to comply with this proposal are likely to be gradual [32],[33]. We view reporting of site-level recruitment to be a logical next step in improving trial transparency, and we hope that many sponsors will help in implementing this initiative.

## Supporting Information

Alternate Language Summary S1 **Translation of the summary points into Spanish, by RD-R.**
(DOC)

Alternate Language Summary S2 **Translation of the summary points into French, by JD-M.**
(DOC)

## Abbreviations

CT: clinical trial
MCT: multicenter clinical trial
REC: research ethics committee
