# Supplementary material for: Disclosure of Investigators' Recruitment Performance in Multicenter Clinical Trials: A Further Step for Research Transparency
Source: PLoS Med. 2011 Dec 27;8(12):e1001149. doi: 10.1371/journal.pmed.1001149 (PMC3246429; doi:10.1371/journal.pmed.1001149)
Supplement: Alternate Language Summary S1 — Translation of the summary points into Spanish, by RD-R. (DOC) [file pmed.1001149.s001.doc]

**Alternate Language Summary S1: Translation of the summary points into** **Spanish**, by Rafael Dal-Ré

Sumario

- Debido a un reclutamiento insuficiente, muchos ensayos finalizan antes de alcanzar el número de casos necesario para comprobar las hipótesis que plantean.

- A través de registros como ClinicalTrial.gov, cualquier persona puede conocer las características más relevantes de un ensayo multicéntrico (EMC)

- Los investigadores de los centros en donde se reclutan los participantes de un ECM son clave para su éxito; sin embargo, la información sobre el reclutamiento de cada centro no es de dominio público.

 - Esta información será de interés para terceros tales como organizaciones de pacientes, promotores y redes de investigadores

- Proponemos que antes de iniciar el ensayo, los promotores informen en ClinicalTrials.gov acerca de los objetivos de reclutamiento de todos los centros implicados, así como del reclutamiento final obtenido en cada uno de ellos. Deberían notificarse también los aspectos que puedan haber influido en el reclutamiento.

- Informar abiertamente de las cifras de reclutamiento de todos los investigadores permitiría a la comunidad científica solicitar al promotor análisis de subgrupos regionales, de forma que se pueda evaluar si las diferencias étnicas o de cuidado estándar pudieran influir en los resultados del tratamiento.
