# Supplementary material for: Disclosure of Investigators' Recruitment Performance in Multicenter Clinical Trials: A Further Step for Research Transparency
Source: PLoS Med. 2011 Dec 27;8(12):e1001149. doi: 10.1371/journal.pmed.1001149 (PMC3246429; doi:10.1371/journal.pmed.1001149)
Supplement: Alternate Language Summary S2 — Translation of the summary points into French, by JD-M. (DOC) [file pmed.1001149.s002.doc]

**Alternate Language Summary S2: Translation of the summary points** **into** **French**, by Jacques Demotes-Mainard

Résumé

- Faute d'un recrutement suffisant, de nombreux essais cliniques sont interrompus avant que le nombre de patients inclus ne permettre de tester les hypothèses sous-tendants les essais. 
- Les registres d'essais cliniques tels que ClinicalTrials.gov permettent à chacun de prendre connaissance des principales caractéristiques des essais cliniques multicentriques (ECM)

- Les sites d'investigation jouent un rôle majeur dans le succès d'un ECM, cependant l'information concernant leur performance en matière de recrutement n'est pas mise à la disposition du public. 
- Nous proposons donc que les promoteurs fournissent sur ClinicalTrials.gov des informations sur les objectifs de recrutement de tous les sites investigateurs avant le début d'un essai, ainsi que sur leur recrutement effectif à la fin de l'essai. Les éléments de nature à interférer avec le recrutement doivent également être décrits. 
- Cette information bénéficiera à l'ensemble des parties prenantes, associations de patients, promoteurs potentiels, réseaux d'essais cliniques.
- La mise à disposition des performances des sites investigateurs en matière de recrutement peut également générer de la part de la communauté scientifique des questions au promoteur sur l'analyse de sous-groupes régionaux, afin d'évaluer si des différences ethniques ou de prise en charge médicale peuvent influer sur l'issue des traitements.
